# Efficacy of the QuitSure App for Smoking Cessation in Adult Smokers: Cross-Sectional Web Survey

**DOI:** 10.2196/49519

**Published:** 2024-05-06

**Authors:** Gregory M Goldgof, Shweta Mishra, Kriti Bajaj

**Affiliations:** 1 Department of Laboratory Medicine University of California, San Francisco San Francisco, CA United States; 2 QuitSure Rapidkart Online Private Limited Mumbai India

**Keywords:** smoking, quit smoking, smoking cessation, smoking app, QuitSure, smoke free, quit vaping, vaping, smoker, smoke, cross-sectional study, smartphone app, tobacco consumption, tobacco, survey, nicotine, nicotine withdrawal, mobile phone

## Abstract

**Background:**

Cigarette smoking remains one of the leading causes of preventable death worldwide. A worldwide study by the World Health Organization concluded that more than 8 million people die every year from smoking, tobacco consumption, and secondhand smoke. The most effective tobacco cessation programs require personalized human intervention combined with costly pharmaceutical supplementation, making them unaffordable or inaccessible to most tobacco users. Thus, digital interventions offer a promising alternative to these traditional methods. However, the leading smartphone apps available in the market today have either not been studied in a clinical setting or are unable to match the smoking cessation success rates of their expensive offline counterparts. We would like to understand whether QuitSure, a novel smoking cessation app built by Rapidkart Online Private Limited, is able to bridge this efficacy gap and deliver affordable and effective smoking cessation at scale.

**Objective:**

Our objective was to do an initial exploration into the engagement, efficacy, and safety of QuitSure based on the self-reported experiences of its users. Outcomes measured were program completion, the effect of program completion on smoking behavior, including self-reported cessation outcomes, and negative health events from using the app.

**Methods:**

All QuitSure registered users who created their accounts on the QuitSure app between April 1, 2021, and February 28, 2022, were sent an anonymized web-based survey. The survey results were added to their engagement data on the app to evaluate the feasibility and efficacy of the app as a smoking cessation intervention. The data were analyzed using descriptive statistics (frequencies and percentages) and the *χ*^2^ test of independence.

**Results:**

In total, 1299 users who had completed the QuitSure program submitted the survey and satisfied the inclusion criteria of the study. Of these, 1286 participants had completed the program more than 30 days before filling out the survey, and 1040 (80.1%, 95% CI 79.1%-82.6%) of them had maintained prolonged abstinence for at least 30 days after program completion. A majority of participants (770/891, 86.4%) who were still maintaining abstinence at the time of submitting the survey did not experience any severe nicotine withdrawal symptoms, while 41.9% (373/891) experienced no mild withdrawal symptoms either. Smoking quantity prior to completing the program significantly affected quit rates (*P*<.001), with heavy smokers (>20 cigarettes per day) having a lower 30-day prolonged abstinence rate (relative risk=0.91; 95% CI 90.0%-96.2%) compared to lighter smokers. No additional adverse events outside of known nicotine withdrawal symptoms were reported.

**Conclusions:**

The nature of web-based surveys and cohort selection allows for extensive unknown biases. However, the efficacy rates of survey respondents who completed the program were high and provide a case for further investigation in the form of randomized controlled trials on the QuitSure tobacco cessation program.

## Introduction

### Background

Cigarette smoking remains one of the leading causes of many premature deaths worldwide [1]. According to the World Health Organization (WHO), more than 8 million people around the world die every year, either directly or indirectly (via secondhand smoke), because of tobacco consumption. Hence, the WHO [2] has identified the tobacco epidemic as one of the world’s biggest public health threats. Beyond the burden of mortality lies the burden of disease as a result of tobacco consumption. For every 1 individual who dies because of smoking, at least 30 live with a serious illness caused by smoking. Smoking causes many health issues, such as cardiovascular diseases, chronic obstructive pulmonary disease, and 12 types of cancer [3]. More than 67% of smokers face debilitating, and eventually fatal, health issues at some point in their smoking lives [4]. Health risks, as well as death risks for smokers compared to nonsmokers, have worsened, due to the deadly spread of COVID-19 across the world [5]. Meanwhile, the economic costs attributable to smoking and exposure to tobacco smoke globally have been estimated to be US $1436 per smoker, which is equivalent to around 1.8% of the world’s gross domestic product [3,6].

In 2015, 68% (22.7 million) of adult smokers said that they wanted to quit smoking. In 2018, 55.1% (21.5 million) of adult smokers said that they had made a quit attempt in the past year. In 2020, 62.5% of youths (middle and high school students) who currently used tobacco products wished to quit all tobacco products, and 65.4% of youths who currently used tobacco products reported that they had stopped using all tobacco products for 1 day or longer in the past year because they were trying to quit [7]. But on the other side of the coin, a report by the National Institute of Cancer, United States [8], found that in 2020, of the 53.9% of smokers who attempted to quit smoking, only 8.5% of them were successful in doing so. In fact, research has found that it takes about 30 quitting attempts for a smoker to successfully quit [9]. The WHO, in 2022, said that without cessation support, only 4% of smokers will be able to successfully quit.

### Smoking Cessation

There are several smoking cessation methods available across the world, including unassisted methods, nicotine replacement therapy (NRT), prescribed medicine (bupropion or varenicline) use, behavioral counseling, quitlines, and the use of mobile apps and websites for smoking cessation [10]. Financial incentives have gained popularity as a cessation method recently [11]. NRT, like nicotine patches, gums, and nasal sprays; medications such as bupropion and varenicline; and nonpharmacological interventions [12] are the most common smoking cessation interventions. However, NRT has shown to have success rates of only 6%-8% [13], while pharmacological interventions, despite their somewhat higher success rates of 14%-20% [14], come with the risk of side effects such as skin irritation or more serious seizures and are also very expensive [15]. Combined interventions for smokers such as behavioral interventions and long-term assistance or social support are most effective when it comes to smoking cessation [15]. However, they tend to be expensive, highly variable depending on the quality of each individual provider, accessible to only small hyperlocal communities, and cannot be scaled up to achieve population-level impact.

### Smartphone Apps for Smoking Cessation

In response to the COVID-19 pandemic, the rate of smoking cessation increased from 23% to 31% [16], which creates the opportunity to encourage and support smokers to quit smoking through different smoking cessation methods. Unconventional methods such as smartphone-based apps can be more useful to increase the odds of quitting success over conventional methods because smartphone use is highly prevalent, is available 24-7, is cost-effective, requires zero-minimal human intervention, and can provide instant and constant support. Seo et al [17] found a total of 603 apps designed for smoking cessation that were available in the US, UK, Australian, and Asian markets [17]. Apps designed for smoking cessation have been downloaded 33 million times globally according to a study done by SensorTower in April 2020 (Nelson, SensorTower.com, personal communication, April 15, 2020). Users who have high engagement with smoking cessation apps have been found to be more likely to be successful in quitting [18,19].

However, literature reviews suggest that only a few apps follow the guidelines for treating tobacco dependence, and most apps use only very simple tools like calculators (41%), calendars (36%), trackers (18%), hypnosis apps (21%), and distractors (10%) [20,21]. According to the WHO, any primary care provider needs to follow the 5As (ask, advise, assess, assist, and arrange) to help a tobacco user [22]. One content analysis study suggested that 96% of the cessation apps addressed “assist” but less frequently addressed the other 4 As [21]. Another review demonstrated that only 11 (6.1%) of the 180 smoking cessation apps available in 2022 have any scientific support [23]. The review also discovered that very few apps offered evidence-based interventions such as mindfulness (18%) or cognitive behavioral therapy (CBT; 2.2%). Other reviews indicated that 88.46% of smoking cessation apps have not been updated by the developers in over a year, and 33.67% of apps have low acceptance by the market with <10,000 downloads [17,21]. Thus, the development of additional smartphone apps that have good user acceptance and are using empirically supported behavior change techniques to deliver smoking cessation interventions appears to be warranted.

### The QuitSure Smoking Cessation Program

The QuitSure program (Rapidkart Online Private Limited) has been identified as one such program, which incorporates behavior change techniques like positive psychology, CBT, and mindfulness that have been shown to be effective in smoking cessation interventions [22,24], and is customized to the smoking habits and psychological needs of the user. It does not include or recommend the use of any pharmaceutical interventions, like oral supplements, medications, or NRTs.

The program follows the 5As recommended by the WHO [22]: it first asks the users about their smoking history such as quantity, patterns, past quitting attempts, and relapse reasons. This also helps the user internalize their smoking behavior. Then, it assesses if they are ready to quit and asks them about their inhibitions to quit. Unless it is a medical condition, it then advises in a clear, strong manner to quit smoking alongside a summary of the program and how it actually works. After understanding the program, willing users are then progressed to the next level, where the app provides the main content on psychology, CBT, and mindfulness [24].

CBT is incorporated by helping users question their beliefs around the positive aspects of smoking and remove them. The app does not demand any lifestyle modification. It simply helps the user accept their smoking triggers and change their response to these triggers. Under mindfulness, the app provides video exercises to teach users how to smoke mindfully by focusing on every aspect of the smoking experience. This exposes to the user the real effects of cigarettes, both while smoking and after, on their bodies and minds. All the content is delivered using empathy and without administering any guilt or blame to the user to keep the user’s mind in a more calm and receptive state.

**Figure 1:**
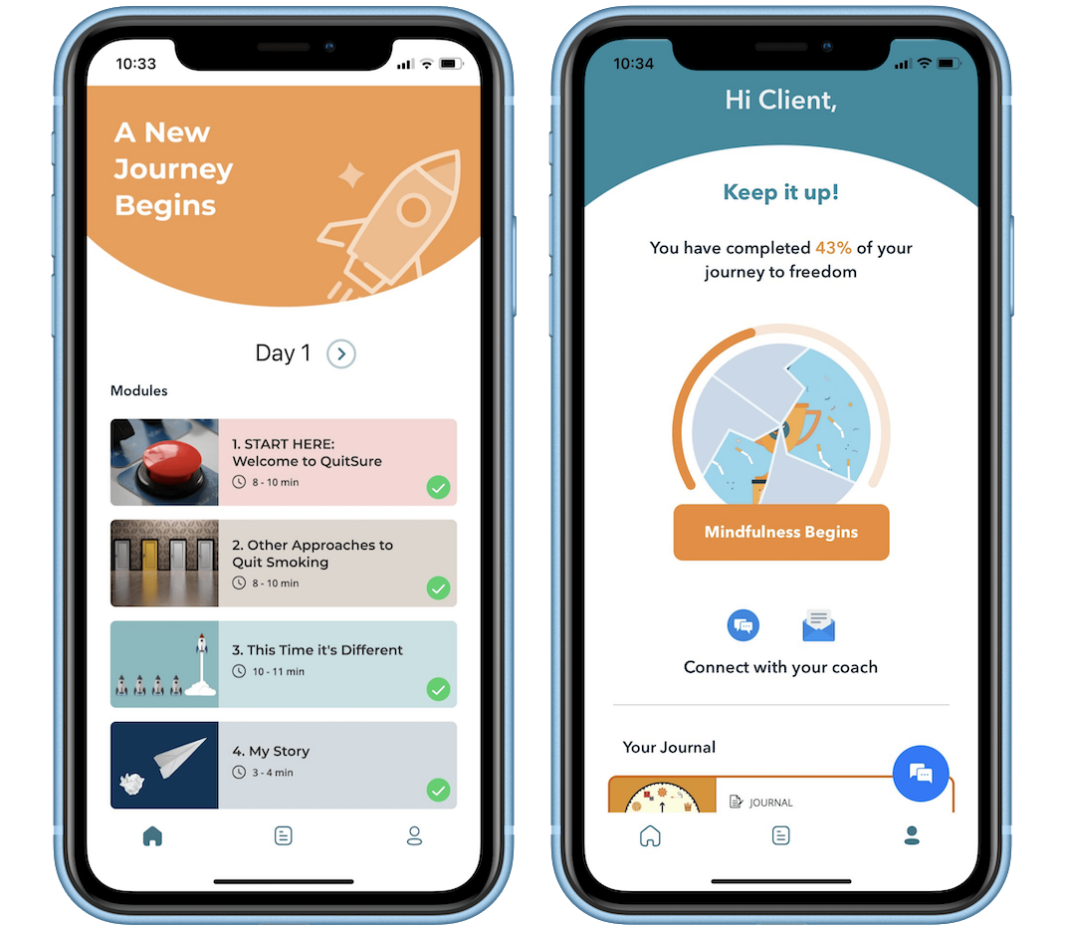
Screenshots of the home page (left) and profile page (right) of the QuitSure app.

Users are required to complete the program in a very specific way with a predefined sequence of content and video exercises as shown in the screenshot of the home page in Figure 1 (left). The program requires around 6-10 hours over 6-12 days to complete. During the whole process, the app has a structured, digital journal for users to record their quitting journey and 24×7 chat-based support from trained counselors for users who have additional questions or concerns that are not addressed by the program itself as shown in Figure 1 (right). Once the user has completed the program and quit smoking, the app has postquit tools and chat support available to prevent them from relapsing. Around 12.3% (4124/33,458) of all users reach out to the counselors for support during and after the program.

The program is priced at US $10 and should be affordable to most daily smokers, as the average global retail price of a pack of 20 cigarettes is approximately US $5 [25,26]. Users only have to pay on day 2 of the 6-day program. This is done, so they can understand how the program is structured and what techniques it uses before committing to it. At the end of the program, users are asked to perform a guided smoking exercise where they smoke their last cigarette and then quit cold turkey. The timestamp when they mark having performed this exercise in the app is considered as their quit date and as their program completion date. The QuitSure app has been updated an average of once every 3 weeks since its launch.

In this paper, we report the results of a retrospective cross-sectional study on users who completed the program between April 1, 2021, and February 28, 2022. Participants who started the QuitSure program but then dropped out before completing it were also surveyed to evaluate the potential areas of improvement for QuitSure. The aim of this study is to evaluate the effectiveness of the QuitSure smoking cessation program to enable smoking cessation among daily smokers. We will also examine the program’s usability, feasibility, and acceptance by the market.

## Methods

### Design

This was a retrospective cross-sectional study to understand, through a web-based survey, smoking cessation outcomes of users who completed the QuitSure program via the QuitSure smartphone app. The survey was conducted in April 2022.

### Recruitment

Users who downloaded and registered for the QuitSure quit smoking app between dates April 1, 2021, and February 28, 2022, and satisfied all the following inclusion criteria were sent the web surveys.

The study included (1) users who were daily smokers as defined by the US Centers for Disease Control and Prevention—smoked at least 1 cigarette per day before they did the program and had smoked at least 100 cigarettes before doing the program [6]; (2) adults aged 18 years and older (self-reported); (3) users who were, at minimum, proficient in the English language (since the program content is solely available in English); (4) users who had completed the entire QuitSure program; (5) and users who had a valid email address.

All registered users of the QuitSure app give consent to be contacted for the purpose of research studies at the time of registration.

### Web-Based Survey

The surveys were created on the web-based Jotform tool built by Jotform Inc. The forms were set up with Jotform’s Health Insurance Portability and Accountability Act (HIPAA) compliant mode, and no personally identifiable data were collected, thus protecting the confidentiality and privacy of the participants. The survey details have been reported in accordance with the Checklist for Reporting Results of Internet E-Surveys (CHERRIES) guidelines [27].

The survey was sent via an automated email from the QuitSure server to all users who fit the inclusion criteria. The email included the relevant details for participation in the study, including the length of the survey, the aim of the study, benefits for the participant, as well as the link to the survey itself. They were encouraged to contact the investigators if they had any questions or concerns. Participation in the survey was voluntary. Participants were granted entry into a lucky draw for a US $50 Amazon voucher. This amount was chosen as being 5 times the price of the QuitSure program, and the email specified that winning the US $50 voucher did not depend on whether their experience with the QuitSure program was positive or negative.

To prevent duplication of data, each email included a hidden, unique, non–personally identifiable ID number that appeared in the survey results. For duplicate submissions, the more recent entry was kept. All questions seen by the participant, depending on the conditions, were mandatory. As a result, incomplete surveys were not recorded or used for analysis. No statistical correction methods, such as weighting of items or propensity scores, were applied to adjust for sample nonrepresentativeness.

The first part of the survey confirmed the participants’ demographic data and satisfaction of the inclusion criteria. It also explicitly requested informed consent for participation in the study. Participants were shown the remaining sections in the survey only if they consented to participate in the study and met the inclusion criteria defined earlier. The second and third parts of each survey are described in the individual sections below. Prior to launching the survey, we conducted usability and technical functionality testing to ensure that participants could easily navigate and complete the electronic questionnaire. In both surveys, the questions were primarily multiple choice, with the rest requiring integer number entries. All multiple choice questions included the option for free-form “other” entries as well as the option for “none” or “choose not to share” as relevant for that question.

### Survey for Program Completers

This survey, referred to as S-Completers, was sent to all users who satisfied the inclusion criteria and had also completed the QuitSure program on the QuitSure app as defined in the introduction. The second section of this survey identified the participants’ smoking history, including the outcome of their completion of the QuitSure program. The third section was conditional. Each participant saw a different set of questions depending on their smoking cessation or reduction or relapse outcome after program completion. The number of questions in each section ranged from 4 to 9 depending on the responses of the participant.

### Survey for Noncompleters of the Program

This survey, referred to as the S-Incompleters survey, was sent to all users who satisfied the inclusion criteria and who had started the program, but then not completed it, to get a qualitative understanding of the feasibility of the program at scale. The S-Incompleters survey also took the participants’ explicit consent for participation in the study and the same demographic data and smoking history questions. The rest of the survey was to understand their reasons for leaving the program midway. The number of questions in each section ranged from 4 to 9 depending on the responses of the participant. The copy of both survey questionnaires is provided in Multimedia Appendices 1 and 2.

### Study Variables

The data collected in the first part of both surveys included demographic information about the participants including gender, age, country of residence, and English proficiency. The second part of both surveys, regarding smoking history and behavior, asked what forms they consumed nicotine in before doing the program, how much and how long they had smoked, and whether they had previously tried to quit smoking using other methods. Those who had completed the QuitSure program were asked for the outcome of their most recent attempt at doing the QuitSure program and if they used any other quit smoking tools, programs, or medications or supplements during or after doing the QuitSure program.

The questions in the third part of the S-Completers survey were dependent on their outcome of doing the QuitSure program. Those who were able to quit 100% since completing the program were asked whether they experienced any mild or severe withdrawal symptoms and weight gain. They were also asked for their current level of cravings to smoke via a Likert scale ranging from 1=none to 5=unbearable. Those who quit for some time, but then relapsed, were asked how long they were able to stay quit and their reasons for relapse. Those who were only able to cut down their smoking level were asked for their new smoking rates and the reasons the program did not help them quit completely. Finally, those for whom the program had no impact, were asked why the program did not work for them. All participants who said they are still smoking were asked their current level of motivation to quit smoking on the Likert scale and whether they would use QuitSure again for their next quitting attempt.

The S-Incompleters survey asked participants why they did not complete the program and whether they were able to quit using a different method since. If they have quit since, then how they quit and what their level of cravings to smoke is. If not, then what their level of motivation to quit is and whether they will use QuitSure as their method of choice.

### Ethical Considerations

This study was approved by the institutional review board of the University of California, San Francisco (IRB 21-35619, reference 331340). The email sent to the users described the study’s aims and procedures as well as the security and confidentiality of their data. It also clearly stated that participation was voluntary, and they could decline to participate. The participants were given a consent form with all the details of the study including the purpose of the study. Participants younger than 18 years were not included in the study. The study observed data protection laws in effect at the time it was conducted. Participants were entered in a lucky draw to win a US $50 gift voucher for Amazon.

### Statistical Analysis

Data were collected from 1299 participants from over 25 countries. The survey responses were available in Microsoft Excel (Microsoft Corp) format. The data analysis tools used were descriptive statistics (frequencies and percentages), pivot tables, as well as chi-square tests of independence.

## Results

### Survey for Program Completers

Of the 13,585 users who were sent the S-Completers survey email, 853 (6.3%) emails bounced. Of the 12,732 delivered emails, 5365 (42.1%) were opened. QuitSure or smoking was not mentioned in the sender or subject lines of the emails, only in the body of the email, to reduce bias based on user perception of the QuitSure program before opening the email. Therefore, we will use this number of email openers as the baseline number of users who were aware of this study. In total, 1906 (35.5%) of those who opened the email clicked on the survey link, and a final 1332 (24.8%) email openers consented to participate in the study and completed the survey. These values are all either equal to or greater than the expected opening (21.5%) and click (8%) rates based on global industry standards [28,29]. Of this set of submitters, 11 were excluded for completing the program in less than 7 days before submitting the survey. An additional 22 were excluded for submitting false data that were significantly different from actual app engagement. A final set of 1299 participants were included in the data analysis for the study. App engagement and preprogram smoking behavior data were not significantly different between those who filled out the survey versus those who did not. A flowchart representing the participant funnel for the S-Completers survey is available in Multimedia Appendix 3.

Table 1 shows the baseline characteristics of the participants, while Multimedia Appendix 4 shows their global distribution. The ratio of male to female participants was found to be 1 to 1.17. The most common age range of participants was found to be 25-34 years (n=431, 33.2%). In total, 97.2% (n=1262) of participants were cigarette smokers, while the remaining 2.6% (n=37) only consumed other forms of nicotine. Five countries, the United States, the United Kingdom, India, Canada, and Australia, had 71.8% (n=933) of the participants.

**Table 1:** Demographic details of participants.

			Male participants (n=587)		Female participants (n=699)		Others (n=13)		All (N=1299)
Age (years), mean (SD)^a^			35.8 (11.74)		41.83 (12.55)		27.11 (6.82)		39.5 (12.56)
Cigarettes smoked per day, mean (SD)			12.57 (8.02)		14.57 (8.56)		13.30 (5.51)		13.66 (8.83)
Median (IQR; smoking category)			10 (7-20; light)		14 (10-20; average)		13 (10-18; average)		12 (8-20; average)
Years smoked, mean (SD)			15.28 (11.40)		21.13 (12.87)		10.15 (9.69)		18.59 (12.62)
**Smoking categories (cigarettes per day), n (%)**									
	Very light (<5)	75 (12.8)		47 (6.7)		0 (0)		122 (9.4)	
	Light (5-10)	119 (20.2)		109 (15.6)		3 (25)		231 (17.8)	
	Average (11-20)	233 (39.6)		301 (43.1)		8 (61.5)		542 (41.7)	
	Heavy (>20)	160 (27.2)		242 (34.6)		2 (15.4)		404 (31.1)	

^a^To calculate the mean, the midpoints of each age group were considered (calculated as upper limit+lower limit/2, ie, 25+35/2 and so on).

Smoking behavior of participants prior to doing the program was grouped into 4 categories as very light (<5 cigarettes per day), light (5-10 cigarettes per day), average (11-20 cigarettes per day), and heavy (>20 cigarettes per day) [30]. The participants smoked an average of 13.66 (SD 8.83) cigarettes per day.

### Effectiveness of the Smartphone App for Smoking Cessation

Participants were divided into 4 overlapping subsets based on the duration between completing the program and submitting the survey. The 4 durations chosen were 7 days, 30 days, 3 months, and 6 months, which are the commonly used smoking cessation milestones [31]. Table 2 shows the self-reported outcome of completing the program for each of these groups. Overall, 88% (1144/1299), 80.9% (1040/1286), 82.4% (991/1203), and 72.4% (725/1002) of participants had maintained prolonged abstinence for 7 days, 30 days, 3 months, and 6 months, respectively. In total, 35 of the 1203 (2.9%) participants were able to cut down on their smoking level in 30 days after the completion of the program, and 19 of the 1002 (1.9%) were able to sustain it for over 6 months after program completion.

**Table 2:** Prolonged abstinence after program completion.

		Participants with 7-day prolonged abstinence (1144/1299, 88%)	Participants with 30-day prolonged abstinence (1040/1286, 80.9%)	Participants with 3-month prolonged abstinence (991/1203, 82.4%)	Participants with 6-month prolonged abstinence (725/1002, 72.4%)
OR^a^ (95% CI)		1.32 (86.8-89.7)	1.20 (79.4-82.3)	1.39 (80-84.6)	1.43 (69.8-75.8)
**Participants by smoking behavior, n/N (%)**					
	Very light (<5)	107/122 (87.7)	95/121 (78.5)	84/101 (83.2)	57/87 (65.5)
	Light (5-10)	209/231 (90.5)	196/228 (86)	183/207 (88.4)	132/173 (76.3)
	Average (11-20)	491/542 (90.6)	446/535 (83.4)	405/466 (86.9)	319/425 (75.1)
	Heavy (>20)	333/404 (82.4)	301/401 (75.1)	271/340 (79.7)	215/316 (68)
**Participants by country, n/N (%)**					
	United States	351/393 (89.3)	324/388 (83.5)	278/357 (77.9)	242/315 (76.8)
	United Kingdom	88/104 (84.6)	73/103 (70.9)	67/97 (69)	56/86 (65)
	India	226/266 (84.6)	195/261 (74.7)	158/231 (68.4)	129/200 (64.5)
	Canada	78/87 (90)	72/86 (83)	58/75 (77)	51/67 (76)
	Australia	68/75 (91)	65/75 (87)	54/67 (81)	50/59 (85)

^a^OR: odds ratio.

The participants who were able to quit smoking as a result of the program and had maintained prolonged cessation at the time of filling out the survey (n=891) experienced varying degrees of withdrawal symptoms, as shown in Tables 3 and 4. Overall, 41.9% (n=373) experienced no mild withdrawal symptoms, and 86.4% (n=770) experienced no severe withdrawal symptoms. In total, 41.9% (n=373) experienced no weight gain after quitting with the QuitSure app; 39.6% (n=353) gained less than 5 kg, while 18.5% (n=165) gained more than 5 kg after quitting.

**Table 3:** Mild withdrawal symptoms reported by participants (n=891).

Symptom	Participants, n (%)
No mild withdrawal symptoms	373 (41.9)
Some mood issues	349 (39.2)
Mild sleep disturbances	196 (22)
Coughing or mild nausea	167 (18.7)
Mild digestive changes	145 (16.3)
Low energy or weakness	143 (16)
Mild headaches	131 (14.7)
Tingling of hands and feet	42 (4.7)
Others	38 (4.3)

**Table 4:** Severe withdrawal symptoms reported by participants (n=891).

Symptom	Participants, n (%)
No severe withdrawal symptoms	770 (86.4)
Increased depression or anxiety	90 (10.1)
Severe headaches or migraines	23 (2.6)
Severe insomnia	22 (2.5)
Severe dizziness or nausea or weakness	19 (2.1)
Strong chest pain	16 (1.8)
Others	10 (1.1)

### Factors Contributing to Success

To assess whether quitting smoking via QuitSure is independent of demographic variables, 2 chi-square tests of independence were conducted as shown in Table 5. The *χ*^2^ value for the impact of gender and age on the efficacy of the program was found to be *χ*^2^_2_=3.8 (*P*=.09) and *χ*^2^_4_=5.9 (*P*=.20), respectively. This indicates that smoking cessation via QuitSure was not dependent on the gender or age of the participants. Smoking behavior prior to starting the program, however, did affect the program’s efficacy. The value was found to be *χ*^2^_1_=20.3 (*P*<.001), indicating a significant impact of smoking behavior on the quit rate. The 30-day prolonged abstinence of heavy smokers was significantly lower than that of those who smoked <20 cigarettes a day (relative risk=0.91; 95% CI 90.0%-96.2%). Country of residence also had a significant impact on program effectiveness with a value of *χ*^2^_4_=9.8 (*P=*.04) when comparing the 5 countries with the most participants. Residents of Australia had the highest 30-day prolonged abstinence rates (relative risk compared to all other participants=1.08, 95% CI 44.0%-83.0%), while residents of the United Kingdom had the lowest (relative risk for 30-day prolonged abstinence compared to all other participants=0.87, 95% CI 84.0%-99.2%).

**Table 5:** Factors affecting smoking cessation rates.

Factor	Chi-square (*df*)	*P* value
Gender	3.8 (2)	.09
Age groups	5.9 (4)	.20
Smoking behavior	20.3 (1)	<.001
Country of residence	9.8 (4)	.04

### Factors Contributing to Failure and Relapse

Table 6 shows the reasons given for failure among participants for whom the program did not work at all (n=35), with fear of quitting (n=15, 42.9%) and lack of belief (n=11, 31.4%) being the most common reasons given. Table 7 shows the major reasons for relapse among participants who quit successfully at first but then relapsed at some point before filling out the survey (n=296). The most likely reasons for relapse were cravings for cigarettes (n=101, 34.1%) and alcohol consumption (n=91, 30.1%).

**Table 6:** Reasons for failure (n=112).

Reason for failure	Participants, n (%)
I was afraid of quitting	15 (42.9)
I did not believe that I could quit	11 (31.4)
I do not know	9 (25.7)
I rushed through the program and may have missed some concepts	7 (20)
I did not do all the steps of the final cigarette transformation ceremony	6 (17.1)
I smoked less than 10 cigarettes mindfully	6 (17.1)
I did not follow all the instructions	5 (14.3)
I took a break >2 days while doing the program	5 (14.3)
I did not believe in the content of the app	2 (5.1)
I did not like the content of the app	2 (5.1)

**Table 7:** Reasons for relapse (n=296).

Reason for relapse	Participants, n (%)
I still had bad cravings and I was unable to resist	101 (34.1)
I gave in while drinking alcohol	91 (30.1)
I faced a tragedy (eg, death of a loved one and bad breakup)	71 (24)
I became overconfident of my success	69 (23.3)
I felt self-destructive	47 (15.9)
I still believe smoking has some benefits	24 (8.1)
I did not do the program properly	18 (6.1)
My physical withdrawal symptoms were very bad	17 (5.7)
I gained a lot of weight	14 (4.7)
Other reasons (stress, peer pressure, etc)	27 (9.1)

Among those who relapsed or were unable to quit after completing the program (n=410), 80.7% (n=331) had a moderate to high motivation to quit at the time of submitting the survey. In total, 91% (n=377) said that they would consider using QuitSure for their next quitting attempt, while 46.1% (n=189) said that they will definitely use QuitSure to quit in the future.

### Survey for Noncompleters of the Program

In total, 19,873 users had dropped off after starting the program and were sent the S-Incompleters survey, of which only 126 (0.6%) consented to participate in the study and submitted the survey.

Table 8 shows the reasons submitted for not completing the program (n=126). The most common reasons given for dropping off midway were a busy schedule (n=51, 40.5%), not enjoying the content of the program or having too much to read (n=25, 19.8%), and lack of belief that the program will work (n=20, 15.9%).

**Table 8:** Reasons for not completing the program (n=126).

Reason for not completing the program	Participants, n (%)
Busy	51 (40.5)
Did not enjoy content or too much reading	25 (19.8)
Lack of belief in the program	20 (15.9)
Quit smoking midway or cut down	16 (12.7)
Had to pay	14 (11.1)
Technical issues	11 (8.7)
Felt program was not working	9 (7.1)
Others	6 (4.7)
Was not ready	5 (4)

## Discussion

### Principal Findings

The purpose of the study was to understand whether the QuitSure program is an effective intervention for smoking cessation and can be implemented at scale to counteract the health and economic consequences of the tobacco epidemic. It was conducted via 2 web-based surveys. In total, 1299 participants submitted the S-Completers survey for program completers. A majority of 80.9% (1040/1286) maintained prolonged abstinence for 30 days after program completion, and 72.4% (725/1002) maintained 6-month prolonged abstinence after program completion. In total, 86.4% (770/891) of participants reported no severe withdrawal symptoms, while 41.9% (373/891) reported no withdrawal symptoms at all. Only 18.5% (165/891) experienced more than 5-kg weight gain after completing the program. Demographic variables such as gender and age did not significantly impact the program’s success, but smoking quantity prior to doing the program and country of residence did have a significant impact on program efficacy. For those who relapsed, cravings and alcohol consumption were major factors, while program noncompletion was attributed to busy schedules or lack of belief in the program by the participants.

The program was able to achieve extended cessation for every category of smoker, from light to heavy with high efficacy rates, and low withdrawal symptoms after quitting. It is easy to navigate and uses simple language. The low price makes it affordable for smokers across most socioeconomic strata, and the easy-to-understand content makes it usable by anyone with a basic understanding of English. The fact that most participants who relapsed, or for whom the program did not work, continue believing in the program’s potential is also a point in its favor. The difference in cessation rates in different countries indicates that the program requires some adaptations to be contextually and culturally relevant to the residents of certain countries.

When it comes to the feasibility of the program to be distributed to the population at large, the dropout rates of the program, at 59.4%, did not show improved program adherence and engagement compared to other health care apps [32]. While the very short 6-day length of the program likely increases completion rates, it requires approximately 1 hour of daily use. This high engagement requirement could be the reason why 40.5% (51/126) of participants who dropped off the program midway state being busy as the reason for noncompletion. The other major reason for dropping off the program was the length and style of the content. QuitSure could break down the program into a 30-day version with just 10-15 minutes of content per day for people who are busy or for whom the content seems too much. They could also add more graphics, videos, and design elements in the content to make it more appealing to users than plain, simple text. Lack of belief in the program’s techniques was also shared as a reason for dropoff. The makers of the app can thus focus on informing the users about the scientific underpinnings of the techniques used in the program as well as include relevant references for their claims throughout the program.

The app is attempting to standardize and replicate an in-person deaddiction counseling program into a do-it-yourself app and uses many of these same psychological tools to achieve success for its users as in-person counseling [33-35]. The efficacy for those who completed the program and participated in the study seems high, indicating some degree of success. However, the program is of the do-it-yourself type and long enough that it requires high self-motivation and high intent to quit on the part of the user to complete the entire program. We do not have data on how many people dropped off even before starting because of the quantity of self-driven work required. A pre-post study analyzing dropout rates at every stage in the user journey will be required to evaluate the true feasibility of the app.

### Limitations

The study had several limitations. A selection bias was created because the base sample selected was solely those who had already signed up for and completed the program, creating a closed cohort and a higher-than-normal intent to quit. Another limitation was the low response rate. Only 24.8% (1332/5365) of those who opened the email chose to submit the survey, allowing for a significant bias toward those for whom the program was successful. If we consider the program to have failed for all those who opened the email but did not submit the survey, the quit rate of QuitSure at the 30-day postprogram time point becomes just 19.4%. A recall bias may have resulted in false memories of withdrawal symptoms during the initial postprogram phase. The single measurement taken eliminates long-term cessation data of participants who only recently completed the program. Finally, the reward for filling out the survey may have motivated participants to give a false-positive response, based on an assumption that it would increase their likelihood of receiving the reward.

Ultimately, the obtained sample is not representative of the smoker population at large. To be able to understand the true feasibility and efficacy of the QuitSure program and counteract the above limitations, we would need to conduct a randomized controlled trial where the self-reported cessation of participants is confirmed via biochemical verification.

### Comparison With Prior Work

Studies have shown that 46.3% of smokers who quit experience significant withdrawal symptoms ranging from anxiety, depression, irritability, and other physical symptoms [36,37]. The biggest strength of the QuitSure program is that only 13.6% (121/891) of the study participants faced severe withdrawal symptoms. Of the remaining participants, only around half faced even the milder withdrawal symptoms such as coughing and mild sleep disturbance, which are usually seen among all smokers upon quitting [38]. This could be a reflection of the program’s focus on the psychological aspect of nicotine addiction via mindfulness, CBT, and reframing mental sets and beliefs, which have previously shown to reduce withdrawal symptoms after quitting [39,40]. Withdrawal symptoms are known to be a key contributor to relapse [36,37]. Therefore, it is likely that the increased effectiveness of the program and higher prolonged cessation rates are a result of these reduced withdrawal symptoms. However, QuitSure does not include any sort of NRT in its protocol. NRT is recommended by the WHO, US Centers for Disease Control and Prevention, as well as the National Institute for Health and Care Excellence, United Kingdom [22,41,42] as an important complement to counseling and has been shown to significantly increase the success rates in psychology-based smoking cessation programs [34,43,44]. The hypothesis given is that NRT reduces withdrawal symptoms and craving levels. QuitSure could include a phased-out nicotine replacement regimen after the program to further increase its efficacy. This is especially important for heavy smokers, for whom the program was less effective.

The primary reason for relapse was due to still experiencing strong cravings for smoking. In fact, 75% (21/28) of participants who experienced greater than moderate levels of cravings after completing the program eventually relapsed. Currently, the QuitSure program does not address cravings management in any specific way after program completion, relying on the program itself to prevent the appearance of cravings at all. To address them and prevent their relapse, QuitSure can monitor craving levels after the program, with additional content and counseling for those who are struggling.

The second reason for relapse was alongside alcohol consumption. The app already recommends users not to drink any alcohol for the first week after quitting. It can extend this further and also give more guidelines on how to handle cravings when drinking alcohol.

Weight gain after quitting is another big concern among smokers, as there is evidence that nicotine reduces appetite, increases metabolism, and reduces food cravings [45,46]. Previous studies have shown that after quitting smoking, 35.4% of quitters had a weight gain over 5% of their body weight [47]. Of the study participants who were able to quit for even a brief period, 58.1% (518/891) had some weight gain. The QuitSure program should do more to specifically address the maladaptive thought patterns and beliefs connecting food, hunger, and smoking.

Previous studies have found that the higher an individual’s app engagement is, the more they are likely to be able to quit smoking [19]. Thus, the QuitSure app needs to improve its engagement rates to increase program completion rates. Some tools the app developers can use to increase engagement that have previously demonstrated success are (1) gamification techniques like leaderboards, progress bars, and levels [19,48,49]; (2) small rewards to participants for every engagement milestone [48]; (3) personalizing notifications and reminders [49]; as well as (4) inclusion of a peer support group to improve program adherence and navigate postquitting withdrawal symptoms and cravings [50].

Overall, within the limitations of the study, the program shows high smoking cessation rates, low rates of withdrawal symptoms and cravings, and a generally positive experience for its users.

### Conclusions

In total, 80.9% (1040/1286) of the survey respondents were able to achieve 30-day prolonged abstinence from smoking after program completion. The program also adheres to the WHO’s 5As guideline for smoking cessation and includes psychological tools used in evidence-based in-person counseling protocols. However, there are many improvements in app engagement, program adherence, and postprogram support that can be made by the app developers. The high success rates, including prolonged cessation rates, among study participants are an indicator that QuitSure could be a useful tool for achieving smoking cessation at scale. Despite the severe limitations and selection biases of the study, the results make the QuitSure program a strong contender for further investigation. Health care institutions should consider and study the program’s feasibility and efficacy in a more controlled setting.

## Appendix

Multimedia Appendix 1 S-Completers survey questions.

Multimedia Appendix 2 S-Incompleters survey questions.

Multimedia Appendix 3 Participant funnel for the S-Completers survey.

Multimedia Appendix 4 Global distribution of participants.

## Abbreviations

CBT: cognitive behavioral therapy
CHERRIES: Checklist for Reporting Results of Internet E-Surveys
HIPAA: Health Insurance Portability and Accountability Act
NRT: nicotine replacement therapy
WHO: World Health Organization

## References

[1] Forouzanfar MH, Alexander L, Anderson HR, Bachman VF, Biryukov S, Brauer M, Burnett R, Casey D, Coates MM, Abd-Allah F, Abera SF, Aboyans V, Abraham B, Abraham JP, Abubakar I, Abu-Rmeileh NME, Aburto TC, Achoki T, Adelekan A, Adofo K, Adou AK, Adsuar JC, Afshin A, Agardh EE, Al Khabouri MJ, Al Lami FH, Alam SS, Alasfoor D, Albittar MI, Alegretti MA, Aleman AV, Alemu ZA, Alfonso-Cristancho R, Alhabib S, Ali R, Ali MK, Alla F, Allebeck P, Allen PJ, Alsharif U, Alvarez E, Alvis-Guzman N, Amankwaa AA, Amare AT, Ameh EA, Ameli O, Amini H, Ammar W, Anderson BO, Antonio CAT, Anwari P, Argeseanu Cunningham Solveig, Arnlöv Johan, Arsenijevic VSA, Artaman A, Asghar RJ, Assadi R, Atkins LS, Atkinson C, Avila MA, Awuah B, Badawi A, Bahit MC, Bakfalouni T, Balakrishnan K, Balalla S, Balu RK, Banerjee A, Barber RM, Barker-Collo SL, Barquera S, Barregard L, Barrero LH, Barrientos-Gutierrez T, Basto-Abreu AC, Basu A, Basu S, Basulaiman MO, Batis Ruvalcaba Carolina, Beardsley J, Bedi N, Bekele T, Bell ML, Benjet C, Bennett DA, Benzian H, Bernabé Eduardo, Beyene TJ, Bhala N, Bhalla A, Bhutta ZA, Bikbov B, Bin Abdulhak Aref A, Blore JD, Blyth FM, Bohensky MA, Bora Başara Berrak, Borges G, Bornstein NM, Bose D, Boufous S, Bourne RR, Brainin M, Brazinova A, Breitborde NJ, Brenner H, Briggs ADM, Broday DM, Brooks PM, Bruce NG, Brugha TS, Brunekreef B, Buchbinder R, Bui LN, Bukhman G, Bulloch AG, Burch M, Burney PGJ, Campos-Nonato IR, Campuzano JC, Cantoral AJ, Caravanos J, Cárdenas Rosario, Cardis E, Carpenter DO, Caso V, Castañeda-Orjuela Carlos A, Castro RE, Catalá-López Ferrán, Cavalleri F, Çavlin Alanur, Chadha VK, Chang JC, Charlson FJ, Chen H, Chen W, Chen Z, Chiang PP, Chimed-Ochir O, Chowdhury R, Christophi CA, Chuang TW, Chugh SS, Cirillo M, Claßen Thomas K D, Colistro V, Colomar M, Colquhoun SM, Contreras AG, Cooper C, Cooperrider K, Cooper LT, Coresh J, Courville KJ, Criqui MH, Cuevas-Nasu L, Damsere-Derry J, Danawi H, Dandona L, Dandona R, Dargan PI, Davis A, Davitoiu DV, Dayama A, de Castro EF, De la Cruz-Góngora Vanessa, De Leo D, de Lima G, Degenhardt L, del Pozo-Cruz B, Dellavalle RP, Deribe K, Derrett S, Des Jarlais DC, Dessalegn M, deVeber GA, Devries KM, Dharmaratne SD, Dherani MK, Dicker D, Ding EL, Dokova K, Dorsey ER, Driscoll TR, Duan L, Durrani AM, Ebel BE, Ellenbogen RG, Elshrek YM, Endres M, Ermakov SP, Erskine HE, Eshrati B, Esteghamati A, Fahimi S, Faraon EJA, Farzadfar F, Fay DFJ, Feigin VL, Feigl AB, Fereshtehnejad SM, Ferrari AJ, Ferri CP, Flaxman AD, Fleming TD, Foigt N, Foreman KJ, Paleo UF, Franklin RC, Gabbe B, Gaffikin L, Gakidou E, Gamkrelidze A, Gankpé Fortuné G, Gansevoort RT, García-Guerra Francisco A, Gasana E, Geleijnse JM, Gessner BD, Gething P, Gibney KB, Gillum RF, Ginawi IAM, Giroud M, Giussani G, Goenka S, Goginashvili K, Gomez Dantes Hector, Gona P, Gonzalez de Cosio Teresita, González-Castell Dinorah, Gotay CC, Goto A, Gouda HN, Guerrant RL, Gugnani HC, Guillemin F, Gunnell D, Gupta R, Gupta R, Gutiérrez Reyna A, Hafezi-Nejad N, Hagan H, Hagstromer M, Halasa YA, Hamadeh RR, Hammami M, Hankey GJ, Hao Y, Harb HL, Haregu TN, Haro JM, Havmoeller R, Hay SI, Hedayati MT, Heredia-Pi IB, Hernandez L, Heuton KR, Heydarpour P, Hijar M, Hoek HW, Hoffman HJ, Hornberger JC, Hosgood HD, Hoy DG, Hsairi M, Hu G, Hu H, Huang C, Huang JJ, Hubbell BJ, Huiart L, Husseini A, Iannarone ML, Iburg KM, Idrisov BT, Ikeda N, Innos K, Inoue M, Islami F, Ismayilova S, Jacobsen KH, Jansen HA, Jarvis DL, Jassal SK, Jauregui A, Jayaraman S, Jeemon P, Jensen PN, Jha V, Jiang F, Jiang G, Jiang Y, Jonas JB, Juel K, Kan H, Kany Roseline Sidibe S, Karam NE, Karch A, Karema CK, Karthikeyan G, Kaul A, Kawakami N, Kazi DS, Kemp AH, Kengne AP, Keren A, Khader YS, Khalifa SEAH, Khan EA, Khang YH, Khatibzadeh S, Khonelidze I, Kieling C, Kim D, Kim S, Kim Y, Kimokoti RW, Kinfu Y, Kinge JM, Kissela BM, Kivipelto M, Knibbs LD, Knudsen AK, Kokubo Y, Kose MR, Kosen S, Kraemer A, Kravchenko M, Krishnaswami S, Kromhout H, Ku T, Kuate Defo Barthelemy, Kucuk Bicer Burcu, Kuipers EJ, Kulkarni C, Kulkarni VS, Kumar GA, Kwan GF, Lai T, Lakshmana Balaji Arjun, Lalloo R, Lallukka T, Lam H, Lan Q, Lansingh VC, Larson HJ, Larsson A, Laryea DO, Lavados PM, Lawrynowicz AE, Leasher JL, Lee JT, Leigh J, Leung R, Levi M, Li Y, Li Y, Liang J, Liang X, Lim SS, Lindsay MP, Lipshultz SE, Liu S, Liu Y, Lloyd BK, Logroscino G, London SJ, Lopez N, Lortet-Tieulent J, Lotufo PA, Lozano R, Lunevicius R, Ma J, Ma S, Machado VMP, MacIntyre MF, Magis-Rodriguez C, Mahdi AA, Majdan M, Malekzadeh R, Mangalam S, Mapoma CC, Marape M, Marcenes W, Margolis DJ, Margono C, Marks GB, Martin RV, Marzan MB, Mashal MT, Masiye F, Mason-Jones AJ, Matsushita K, Matzopoulos R, Mayosi BM, Mazorodze TT, McKay AC, McKee M, McLain A, Meaney PA, Medina C, Mehndiratta MM, Mejia-Rodriguez F, Mekonnen W, Melaku YA, Meltzer M, Memish ZA, Mendoza W, Mensah GA, Meretoja A, Mhimbira FA, Micha R, Miller TR, Mills EJ, Misganaw A, Mishra S, Mohamed Ibrahim Norlinah, Mohammad KA, Mokdad AH, Mola GL, Monasta L, Montañez Hernandez Julio C, Montico M, Moore AR, Morawska L, Mori R, Moschandreas J, Moturi WN, Mozaffarian D, Mueller UO, Mukaigawara M, Mullany EC, Murthy KS, Naghavi M, Nahas Z, Naheed A, Naidoo KS, Naldi L, Nand D, Nangia V, Narayan KMV, Nash D, Neal B, Nejjari C, Neupane SP, Newton CR, Ngalesoni FN, Ngirabega Jean de Dieu, Nguyen G, Nguyen NT, Nieuwenhuijsen MJ, Nisar MI, Nogueira JR, Nolla JM, Nolte S, Norheim OF, Norman RE, Norrving B, Nyakarahuka L, Oh IH, Ohkubo T, Olusanya BO, Omer SB, Opio JN, Orozco R, Pagcatipunan RS, Pain AW, Pandian JD, Panelo CIA, Papachristou C, Park EK, Parry CD, Paternina Caicedo Angel J, Patten SB, Paul VK, Pavlin BI, Pearce N, GBD 2013 Risk Factors Collaborators (2015). Global, regional, and national comparative risk assessment of 79 behavioural, environmental and occupational, and metabolic risks or clusters of risks in 188 countries, 1990-2013: a systematic analysis for the Global Burden of Disease Study 2013. Lancet.

[2] (2021). Tobacco. World Health Organization.

[3] (2022). Economic trends in tobacco. Centers for Disease Control and Prevention.

[4] (2022). More than 100 reasons to quit tobacco. Pan American Health Organization.

[5] Alqahtani JS, Oyelade T, Aldhahir AM, Alghamdi SM, Almehmadi M, Alqahtani AS, Quaderi S, Mandal S, Hurst JR (2020). Prevalence, severity and mortality associated with COPD and smoking in patients with COVID-19: a rapid systematic review and meta-analysis. PLoS One.

[6] Goodchild M, Nargis N, Tursan d'Espaignet E (2018). Global economic cost of smoking-attributable diseases. Tob Control.

[7] Babb S, Malarcher A, Schauer G, Asman K, Jamal A (2017). Quitting smoking among adults—United States, 2000-2015. MMWR Morb Mortal Wkly Rep.

[8] (2022). Quitting smoking. National Cancer Institute.

[9] Chaiton M, Diemert L, Cohen JE, Bondy SJ, Selby P, Philipneri A, Schwartz R (2016). Estimating the number of quit attempts it takes to quit smoking successfully in a longitudinal cohort of smokers. BMJ Open.

[10] Lund M, Kvaavik E (2021). Methods used in smoking cessation and reduction attempts: findings from help-seeking smokers. J Smok Cessat.

[11] Notley C, Gentry S, Livingstone-Banks J, Bauld L, Perera R, Hartmann-Boyce J (2019). Incentives for smoking cessation. Cochrane Database Syst Rev.

[12] Fiore MC, Jaén CR, Baker TB, Bailey WC, Benowitz NL, Curry SJ, Dorfman SF, Froelicher ES, Goldstein MG, Healton CG, Henderson PN, Heyman RB, Koh HK, Kottke TE, Lando HA, Mecklenburg RE, Mermelstein RJ, Mullen PD, Orleans CT, Robinson L, Stitzer ML, Tommasello AC, Villejo L, Wewers ME (2008). Treating tobacco use and dependence: 2008 update. US Department of Health and Human Services, Public Health Service.

[13] Etter JF, Stapleton JA (2006). Nicotine replacement therapy for long-term smoking cessation: a meta-analysis. Tob Control.

[14] Cahill K, Stevens S, Perera R, Lancaster T (2013). Pharmacological interventions for smoking cessation: an overview and network meta-analysis. Cochrane Database Syst Rev.

[15] Stitzer ML (1999). Combined behavioral and pharmacological treatments for smoking cessation. Nicotine Tob Res.

[16] Kayhan Tetik B, Gedik Tekinemre I, Taş S (2021). The effect of the COVID-19 pandemic on smoking cessation success. J Community Health.

[17] Seo S, Cho SI, Yoon W, Lee CM (2022). Classification of smoking cessation apps: quality review and content analysis. JMIR Mhealth Uhealth.

[18] Zeng EY, Heffner JL, Copeland WK, Mull KE, Bricker JB (2016). Get with the program: adherence to a smartphone app for smoking cessation. Addict Behav.

[19] Browne J, Halverson TF, Vilardaga R (2021). Engagement with a digital therapeutic for smoking cessation designed for persons with psychiatric illness fully mediates smoking outcomes in a pilot randomized controlled trial. Transl Behav Med.

[20] Abroms LC, Lee Westmaas J, Bontemps-Jones J, Ramani R, Mellerson J (2013). A content analysis of popular smartphone apps for smoking cessation. Am J Prev Med.

[21] Hoeppner BB, Hoeppner SS, Seaboyer L, Schick MR, Wu GWY, Bergman BG, Kelly JF (2016). How smart are smartphone apps for smoking cessation? A content analysis. Nicotine Tob Res.

[22] (2014). Toolkit for delivering the 5A’s and 5R’s brief tobacco interventions in primary care. World Health Organization.

[23] Bold KW, Garrison KA, DeLucia A, Horvath M, Nguyen M, Camacho E, Torous J (2023). Smartphone apps for smoking cessation: systematic framework for app review and analysis. J Med Internet Res.

[24] Hersi M, Traversy G, Thombs BD, Beck A, Skidmore B, Groulx S, Lang E, Reynolds DL, Wilson B, Bernstein SL, Selby P, Johnson-Obaseki S, Manuel D, Pakhale S, Presseau J, Courage S, Hutton B, Shea BJ, Welch V, Morrow M, Little J, Stevens A (2019). Effectiveness of stop smoking interventions among adults: protocol for an overview of systematic reviews and an updated systematic review. Syst Rev.

[25] (2023). Price rankings by country of cigarettes 20 pack (Marlboro) (markets). Numbeo.

[26] Darden ME, Hotchkiss JL, Pitts MM (2020). Where there's smoke…: the wage impact of smoking. Federal Reserve Bank Atlanta's Policy Hub.

[27] Eysenbach G (2004). Improving the quality of web surveys: the Checklist for Reporting Results of Internet E-Surveys (CHERRIES). J Med Internet Res.

[28] (2022). What are good open rates, CTRs, and CTORs for email campaigns?. Campaign Monitor.

[29] Chung L What is a good survey response rate for online customer surveys?. Delighted.

[30] DeVita VT (2023). How do I know if I’m a light, average, or heavy smoker?. ShareCare.

[31] Japuntich SJ, Leventhal AM, Piper ME, Bolt DM, Roberts LJ, Fiore MC, Baker TB (2011). Smoker characteristics and smoking-cessation milestones. Am J Prev Med.

[32] Torous J, Lipschitz J, Ng M, Firth J (2020). Dropout rates in clinical trials of smartphone apps for depressive symptoms: a systematic review and meta-analysis. J Affect Disord.

[33] Manjula DC, Shekar V, Reddy RCJ (2018). Effect of behavioural counselling in tobacco cessation. J Sci Dent.

[34] Lindson-Hawley N, Thompson TP, Begh R (2015). Motivational interviewing for smoking cessation. Cochrane Database Syst Rev.

[35] Blebil AQ, Sulaiman SAS, Hassali MA, Dujaili JA, Zin AM (2014). Impact of additional counselling sessions through phone calls on smoking cessation outcomes among smokers in Penang State, Malaysia. BMC Public Health.

[36] McLaughlin I, Dani JA, De Biasi M (2015). Nicotine withdrawal. Curr Top Behav Neurosci.

[37] Lancaster T, Stead LF (2017). Individual behavioural counselling for smoking cessation. Cochrane Database Syst Rev.

[38] Thornley S, McRobbie H, Lin RB, Bullen C, Hajek P, Laugesen M, Senior H, Whittaker R (2009). A single-blind, randomized, crossover trial of the effects of a nicotine pouch on the relief of tobacco withdrawal symptoms and user satisfaction. Nicotine Tob Res.

[39] Cui ZY, Li YH, Liu Z, Li L, Nie XQ, Zhou XM, Cheng AQ, Li JX, Qin R, Wei XW, Zhao L, Ladmore D, Pesola F, Chung KF, Chen ZM, Hajek P, Xiao D, Wang C (2022). The experience of tobacco withdrawal symptoms among current smokers and ex-smokers in the general population: findings from nationwide China Health Literacy Survey during 2018-19. Front Psychiatry.

[40] Mhende J, Bell SA, Cottrell-Daniels C, Luong J, Streiff M, Dannenfelser M, Hayat MJ, Spears CA (2021). Mobile delivery of mindfulness-based smoking cessation treatment among low-income adults during the COVID-19 pandemic: pilot randomized controlled trial. JMIR Form Res.

[41] (2023). Quit smoking. Centers for Disease Control and Prevention.

[42] (2023). Recommendations on promoting quitting. National Institute for Health and Care Excellence.

[43] Jódar-Sánchez F, Carrasco Hernández L, Núñez-Benjumea FJ, Mesa González MA, Moreno Conde J, Parra Calderón CL, Fernandez-Luque L, Hors-Fraile S, Civit A, Bamidis P, Ortega-Ruiz F (2018). Using the Social-Local-Mobile app for smoking cessation in the SmokeFreeBrain project: protocol for a randomized controlled trial. JMIR Res Protoc.

[44] Carrasco-Hernandez L, Jódar-Sánchez F, Núñez-Benjumea F, Moreno Conde J, Mesa González M, Civit-Balcells A, Hors-Fraile S, Parra-Calderón CL, Bamidis PD, Ortega-Ruiz F (2020). A mobile health solution complementing psychopharmacology-supported smoking cessation: randomized controlled trial. JMIR Mhealth Uhealth.

[45] Chen H, Hansen MJ, Jones JE, Vlahos R, Bozinovski S, Anderson GP, Morris MJ (2006). Cigarette smoke exposure reprograms the hypothalamic neuropeptide Y axis to promote weight loss. Am J Respir Crit Care Med.

[46] Bloom EL, Farris SG, DiBello AM, Abrantes AM (2019). Smoking-related weight and appetite concerns and use of electronic cigarettes among daily cigarette smokers. Psychol Health Med.

[47] Jeremias-Martins E, Chatkin JM (2019). Does everyone who quit smoking gain weight? A real-world prospective cohort study. J Bras Pneumol.

[48] Garaialde D, Cox AL, Cowan BR (2021). Designing gamified rewards to encourage repeated app selection: effect of reward placement. Int J Hum-Comput Stud.

[49] Jakobsen PR, Christensen JR, Nielsen JB, Søndergaard J, Jarbøl DE, Olsen MH, Nielsen JS, Kristensen JK, Brandt CJ (2021). Identification of important factors affecting use of digital individualised coaching and treatment of type 2 diabetes in general practice: a qualitative feasibility study. Int J Environ Res Public Health.

[50] Cheung YTD, Chan CHH, Lai CKJ, Chan WFV, Wang MP, Li HCW, Chan SSC, Lam TH (2015). Using WhatsApp and Facebook online social groups for smoking relapse prevention for recent quitters: a pilot pragmatic cluster randomized controlled trial. J Med Internet Res.

