# Supplementary material for: Efficacy of the QuitSure App for Smoking Cessation in Adult Smokers: Cross-Sectional Web Survey
Source: JMIR Hum Factors. 2024 May 6;11:e49519. doi: 10.2196/49519 (PMC11106700; doi:10.2196/49519)
Supplement: Multimedia Appendix 1 [file humanfactors_v11i1e49519_app1.pdf]

A. Informed Consent

1. I am... ______ years old

[1] <18 yrs old
[2] 18-24
[3] 25-34
[4] 35-44
[5] 45-59
[6] 60-74
[7] >75 years old

2. My English language ability is.... "[1] Fluent (Native)

[2] Fluent (Non-Native)
[3] Proficient
[4] Conversational
[5] Basic
[6] None

3. The country I currently live in is....

4. I identify as....

[1] Male
[2] Female
[3] Other

5. My relationship with QuitSure:

[1] I have no relationship with QuitSure or its team beyond the scope of this program
[2] I work with QuitSure or one of its subsidiaries/partners/collaborators
[3] I know the creators of QuitSure personally"

6. I have a clinically diagnosed mental health disorder "[1] No

[2] Yes, but it is mild/being treated (e.g. mild depression, mild anxiety, etc)
[3] Yes, and it is severe/unmanaged (e.g. severe PTSD, self-harm, schizophrenia, etc)"

I consent to having my de-identified (or anonymous) data entered or shown in this survey shared with researchers for the purpose of this study.

Your individual details will never be disclosed. No identifiable data will be shared with the researchers. Only aggregated data will be published in the study.
[1] Yes
[2] No

B. My Smoking History -

1. Before doing the QuitSure program, I used to smoke....

[1] Cigarettes only
[2] Vapes only
[3] Both Cigarettes and Vapes
[4] I only consumed smokeless tobacco (e.g. chewing, dipping, etc)

2. Before trying QuitSure I had tried to quit smoking using .... (check all that apply)

[1] Cold Turkey
[2] Cutting Down
[3] Nicotine gums/patches
[4] Medications (e.g. Champix, Wellbutrin, etc)
[5] Vaping
[6] Hypnotherapy
[7] In-person psychological program/consultations
[8] Book or Video Program (e.g. EasyWay, WhyQuit, etc)
[9] A mobile app (non-QuitSure)
[10] Other ______________________

3. My QuitSure quit date (when I smoked my final cigarette via the QuitSure program) was approximately....

4. If you have multiple quit dates via QuitSure, put your most recent one.

5. I did the QuitSure Program

[1] Just once
[2] Twice
[3] More than twice

6. When I completed the QuitSure Program (most recently), I….

[1] Quit smoking completely and I am still a non smoker
[2] Quit smoking for some time but then relapsed and started smoking again
[3] Was only able to cut down smoking and I still smoke less than I did before
[4] Was able to to cut down smoking for a short while but now I am back to my original levels of smoking
[5] Was not able to change my smoking habits at all

"This section is only shown to successful quitters
(They chose option 1 in question 2.5 above)" -

7. After I quit smoking, I experienced some mild withdrawal symptoms like....

[1] No withdrawal symptoms
[2] Mild sleep disturbance
[3] Coughing/mild nausea

[4] Mild digestive changes (e.g. constipation)
[5] Mild headaches
[6] Some mood issues, e.g. irritability, anger, etc
[7] Low energy/weakness for a few days
[8] Tingling of hands and feet
[9] Other(s) _______________

8. After I quit smoking, I experienced some severe withdrawal symptoms like....

[1] No severe withdrawal symptoms
[2] Increased depression or anxiety
[3] Severe headaches/migraines
[4] Severe insomnia
[5] Strong chest pain
[6] Severe dizziness/nausea/weakness
[7] Other(s) _____________

9. After I quit smoking, my weight....

[1] Reduced
[2] Remained the same
[3] Increased a bit
[4] Increased more than 5kgs/10lbs

10. My current level of craving to smoke is.....

[1] Minimal/None
[2] Mild
[3] Moderate
[4] High
[5] Unbearable

This section is shown to successful quitters who relapsed after some time (They chose option 2 in question 2.5 above) -

11. I was able to stay smoke-free for _______________ days

12. I relapsed because.....(check all that apply)

[1] I became overconfident of my success
[2] I still had bad cravings and I was unable to resist
[3] My physical withdrawal symptoms were very bad
[4] I gave in while drinking alcohol
[5] I still believe smoking has some benefits
[6] I gained a lot of weight
[7] I did not do the program properly

[8] I felt self-destructive
[9] I faced a tragedy (e.g. death of a loved one, bad breakup, etc)
[10] Other ____________

"This section is only shown to those who were only able to cut down, not quit (They chose option 3 or 4 in question 2.5 above)" -

13. How much were you able to cut down your smoking? I was able to cut down to ..... ___________ cigarettes on average per week
14. How much were you able to cut down your smoking right when you finished the program? I was able to cut down to ..... ___________ cigarettes on average per week
15. For how long were you able to cut down your smoking? _______________ days
16. I think I was unable to quit smoking completely using QuitSure because...(check all that apply)

[1] I didn't want to quit completely, I am happy with just cutting down
[2] I rushed through the program and may have missed some concepts
[3] I faced withdrawal symptoms
[4] I did not believe that I could quit
[5] I was afraid of quitting completely
[6] I did not believe all the content in the app
[7] I did not like the content in the app
[8] Other ________________

"This section is only shown to clients who were not able to quit at all via the program (They chose option 5 in question 2.5 above)" -

17. I think the QuitSure program did not work for me because...(check all that apply)

[1] I rushed through the program and may have missed some concepts
[2] I smoked less than 10 cigarettes mindfully
[3] I took a break for >2 days while doing the program
[4] I did not follow all the instructions
[5] I did not do all the steps of the final cigarette transformation ceremony
[6] I did not believe that I could quit
[7] I was afraid of quitting
[8] I did not believe the content in the app
[9] I did not like the content in the app
[10] Other ______________

"This section is followup questions for all clients who are still smoking (They come here from sections 4, 5, and 6. They chose option 2, 3, 4, or 5 in question 2.5 above)" -

18. My current level of motivation to quit smoking is...

[1] High motivation
[2] Moderately motivated
[3] Low/Mild motivation
[4] Not at all motivated to quit smoking

19. If I attempt to quit smoking again in the future

[1] I will definitely use QuitSure
[2] I may use QuitSure
[3] I will definitely NOT use QuitSure

"Shown to everyone
(They come here from sections 3 and 7)"   -

20. During and/or after doing the QuitSure Program, I also used other quitting techniques to increase my chances of success

[1] No, none
[2] Nicotine gums/patches
[3] Medications (e.g. Champix, Wellbutrin, etc)
[4] Hypnotherapy
[5] In-person psychological program
[6] Book or Video Program (e.g. EasyWay, WhyQuit, etc)
[7] A mobile app (non-QuitSure)
[8] Other ______________________"

Conclusion: (Thank you and goodbye messaging)"   -
"Thank you for participating in our study!
