# Supplementary material for: Efficacy of the QuitSure App for Smoking Cessation in Adult Smokers: Cross-Sectional Web Survey
Source: JMIR Hum Factors. 2024 May 6;11:e49519. doi: 10.2196/49519 (PMC11106700; doi:10.2196/49519)
Supplement: Multimedia Appendix 2 [file humanfactors_v11i1e49519_app2.pdf]

A. Informed Consent -

1. I am... ______ years old

2. My English language ability is....

[1] Fluent (Native)
[2] Fluent (Non-Native)
[3] Proficient
[4] Conversational
[5] Basic
[6] None

3. The country I currently live in is....

4. I identify as....

[1] Male
[2] Female
[3] Other"

5. My relationship with QuitSure

[1] I have no relationship with QuitSure or its team beyond the scope of this program
[2] I work with QuitSure or one of its subsidiaries/partners/collaborators
[3] I know the creators of QuitSure personally"

6. I have a clinically diagnosed mental health disorder

[1] No
[2] Yes, but it is mild/being treated (e.g. mild depression, mild anxiety, etc)
[3] Yes, and it is severe/unmanaged (e.g. severe PTSD, self-harm, schizophrenia, etc)"

7. “I consent to having my de-identified (or anonymous) data entered or shown in this survey shared with researchers for the purpose of this study. Your individual details will never be disclosed. No identifiable data will be shared with the researchers. Only aggregated data will be published in the study.”

[1] Yes
[2] No"

B. My Smoking History -

8. Before doing the QuitSure program, I used to smoke....

[1] Cigarettes only
[2] Vapes only
[3] Both
[4] I only consumed smokeless tobacco (e.g. chewing, dipping, etc)

9. Other techniques I have used to try and quit smoking are...

[1] Cold Turkey

[2] Cutting Down
[3] Nicotine gums/patches
[4] Medications (e.g. Champix, Wellbutrin, etc)
[5] Vaping
[6] Hypnotherapy
[7] In-person psychological program/consultations
[8] Book or Video Program (e.g. EasyWay, WhyQuit, etc)
[9] A mobile app (non-QuitSure)
[10] Other ____________________

C. My Engagement with QuitSure -

10. I left the QuitSure program midway because

[1] I quit smoking midway through the program itself
[2] I cut down and was happy with my new level of smoking
[3] I was busy and did not have time to continue
[4] The program had too much reading
[5] I did not enjoy the content in the program
[6] There were technical issues in the app
[7] I did not believe that the app would work
[8] Other ____________

11. I currently primarily (choose the one that applies best)

[1] Smoke cigarettes
[2] Vape
[3] Consume smokeless tobacco (e.g. chewing, dipping, etc)
[4] Have quit and no longer consume tobacco/nicotine products"

"Only shown to those who have quit
(Selected option 4 in question 3.2 above)" -

12. I was able to successfully quit smoking/tobacco by using

[1] The QuitSure Program
[2] Self-control / will-power
[3] Nicotine gums/patches
[4] Medications (e.g. Champix, Wellbutrin, etc)
[5] Vaping
[6] Hypnotherapy
[7] In-person psychological program/consultations
[8] Book or Video Program (e.g. EasyWay, WhyQuit, etc)
[9] A mobile app (non-QuitSure)
[10] Other ____________________

13. My current level of craving to smoke is.....

[1] Minimal/None
[2] Mild
[3] Moderate
[4] High
[5] Unbearable"

"Only shown to those who are still smoking
(Selected option 1, 2, or 3 in question 3.2 above)" -

14. My current level of motivation to quit smoking is...

[1] High motivation
[2] Moderately motivated
[3] Low/Mild motivation
[4] Not at all motivated to quit smoking

15. If I attempt to quit smoking again in the future

[1] I will definitely use QuitSure
[2] I may use QuitSure
[3] I will definitely NOT use QuitSure"

"Conclusion" (Thank you and goodbye messaging)" **-**
