# Supplementary figures and images for: Efficacy of the QuitSure App for Smoking Cessation in Adult Smokers: Cross-Sectional Web Survey
Source: JMIR Hum Factors. 2024 May 6;11:e49519. doi: 10.2196/49519 (PMC11106700; doi:10.2196/49519)

Recruitment

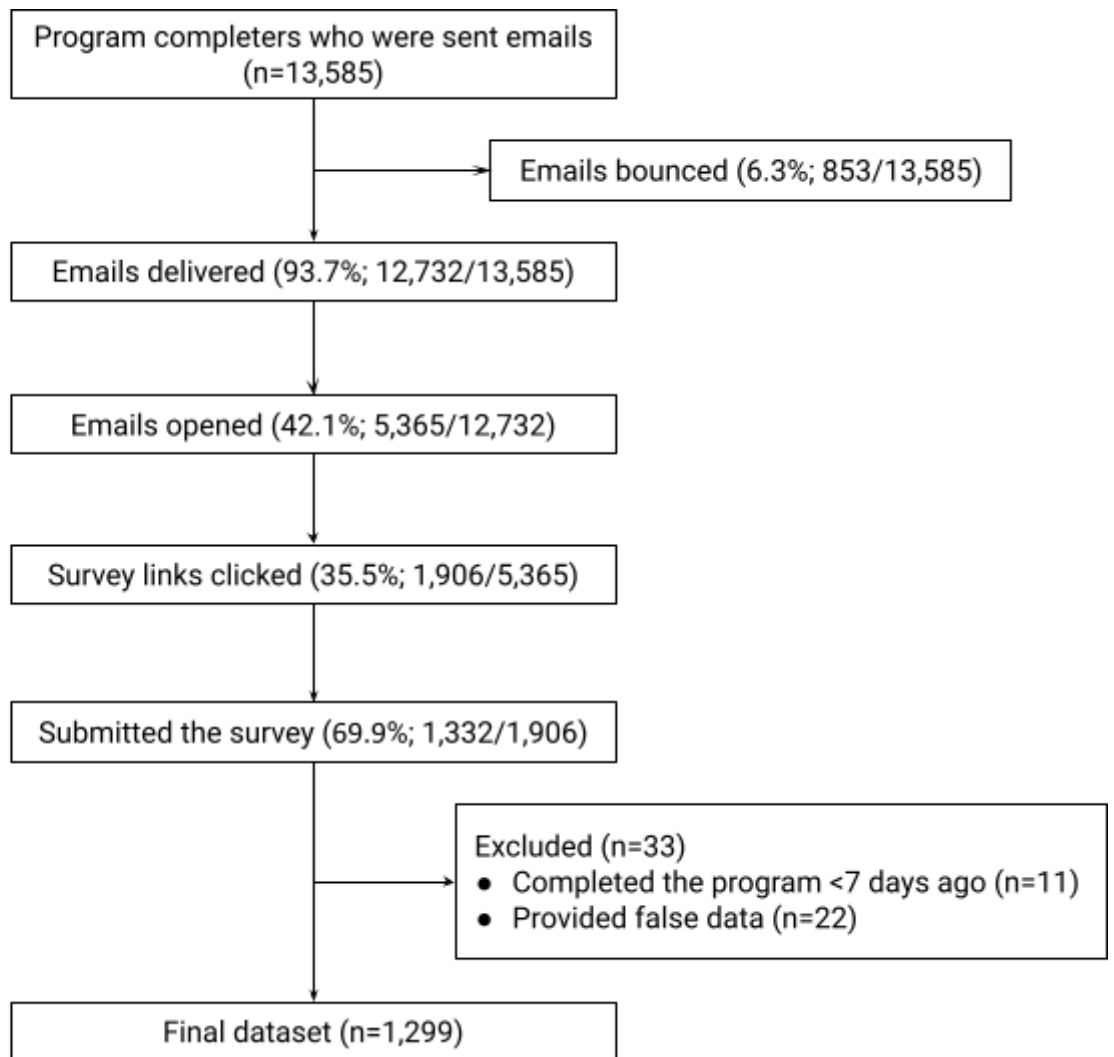

Supplement: Multimedia Appendix 3 [file humanfactors_v11i1e49519_app3.pdf]
