# Supplementary material for: Efficacy of the QuitSure App for Smoking Cessation in Adult Smokers: Cross-Sectional Web Survey
Source: JMIR Hum Factors. 2024 May 6;11:e49519. doi: 10.2196/49519 (PMC11106700; doi:10.2196/49519)
Supplement: Multimedia Appendix 4 [file humanfactors_v11i1e49519_app4.pdf]

Countries	*FEMALE*	*MALE*	*OTHERS*	*ALL*
United States	301	88	4	393
United Kingdom	73	31	0	104
India	22	244	0	266
Australia	59	15	1	75
Canada	60	24	3	87
Albania	1	1	0	2
Austria	4	1	0	5
Bangladesh	0	7	0	7
Belgium	3	8	1	12
Brazil	0	2	0	2
Croatia	9	3	0	12
Czech Republic	1	4	1	6
Denmark	1	2	1	4
Egypt	1	2	0	3

Estonia	2	1	0	3
Ethiopia	0	1	0	1
eSwatini	1	0	0	1
Finland	1	0	0	1
France	10	4	0	14
Germany	15	11	0	26
Greece	2	7	0	9
Hungary	1	2	0	3
Indonesia	1	0	0	1
Ireland	1	9	0	26
Israel	1	3	0	4
Italy	9	12	0	21
Jamaica	2	0	0	2
Kenya	0	1	0	1
Latvia	0	1	0	1
Lithuania	1	0	0	1
Malawi	0	1	0	1

Malaysia	1	4	0	5
Malta	3	0	0	3
Mexico	1	4	0	5
Morocco	2	0	0	2
Nepal	0	1	0	1
Netherlands	9	5	0	14
New Zealand	9	5	0	14
Nigeria	0	3	0	3
Norway	1	0	0	1
Pakistan	1	4	0	5
Papua New Guinea	0	2	0	2
Peru	0	1	0	1
Philippines	7	13	0	20
Poland	2	1	0	3
Portugal	3	2	0	5
Qatar	0	3	0	3

Romania	7	5	0	12
Singapore	1	2	0	3
Slovakia	1	0	0	1
Slovenia	4	3	0	7
South Africa	35	33	2	70
South Korea	0	1	0	1
Spain	4	0	0	4
Switzerland	4	3	0	7
Trinidad and Tobago	1	0	0	1
Tunisia	1	0	0	1
Turkey	3	2	0	5
United Arab Emirates	0	1	0	1
Venezuela	0	1	0	1
Vietnam	0	1	0	1
Zimbabwe	1	3	0	4
Grand Total	699	588	13	1299
